# ICD-11 Burnout for the psychiatrist: Meaning of the concept and prevalence of the condition.

**DOI:** 10.1192/j.eurpsy.2024.321

**Published:** 2024-08-27

**Authors:** J. M. Pelayo-Terán, Z. Gutiérrez-Hervás, S. Vega-García, M. E. García-Llamas, C. López-Zapico, Y. Zapico-Merayo

**Affiliations:** ^1^Servicio de Psiquiatría y Salud Mental; ^2^Unidad de Calidad y Seguridad del Paciente, Hospital El Bierzo. Gerencia de Asistencia Sanitaria del Bierzo (GASBI). SACYL, Ponferrada; ^3^Área de Medicina Preventiva y Salud Pública. Departamento de Ciencias Biomédicas, Universidad de León, León; ^4^Grupo CB07/09/2001, Centro de Investigación Biomédica en Red de Salud Mental (CIBERSAM), Madrid; ^5^Facultad de Ciencias Jurídicas y Sociales, Universidad Juan Carlos I, Móstoles, Madrid, Spain

## Abstract

**Introduction:**

Burnout was reclassified in 2019 as an occupational phenomenon in ICD-11. The new condition includes the classic tridimensional definition with symptoms in areas of fatigue/energy depletion, mental distance/cinism and sense of ineffectiveness/lack of accomplishment.

**Objectives:**

To evaluate the knowledge and perceptions of psychiatrists regarding new ICD-11 burnout definition.

To analyse the frequency of burnout symptoms in the psychiatric consultations and among the psychiatrists as healthcare professionals.

**Methods:**

An online survey (designed with Microsoft® *Forms*) was sent in June 2023 to psychiatrists from three regions of Spain, contacted form local scientific societies. Psychiatrists, currently working, had to consent and answer a brief survey (average time: 2 min 32 sec) of 9 questions regarding the definition of burnout, their experience in clinical practice, their own symptoms and symptoms observed in colleagues.

**Results:**

164 psychiatrists answered, 114 females (69.5%), mean age: 43.61 ± 11.28 years. 48.2% assured they had never used the term Burnout or the ICD codes Z73.0/QD85, whereas a 9.1% used them frequently in clinical practice. 58.5% considered burnout just a condition related to work and a 38.4% either a syndrome or a disorder.

Most psychiatrists referred that their patients exhibited symptoms of the three dimensions. Fatigue was the most common, attended frequently by 79.5% of the surveyed, followed by ineffectiveness (73.1%) and cinism (65.3%).

When reporting their own symptoms, only 16.5% psychiatrists referred not suffering any symptom. The most frequently involved was fatigue (66.5%), then ineffectiveness (56.1%) and cinism (41.5%). 28,7% reported concomitant symptoms of the three dimensions.

70.7% recognized fatigue symptoms in their colleagues, 61% ineffectiveness, 72.6% cinism and 45,5% recognized symptoms from the three dimensions. Only a 7.3% did not identify any of them.

A younger age was related to higher probability of suffering cinism (T:2.546; p=0.012) and ineffectiveness (T:2.900; p=0.004) and to a higher probability of recognizing cinism (T=3,293; p=0,001) an ineffectiveness in others (T=2.355; p=0.020)

Females showed a higher frequency of ineffectiveness symptoms (61.4% vs 44%; χ^2^:4.274; p=0,029).

**Image:**

**Figure:**
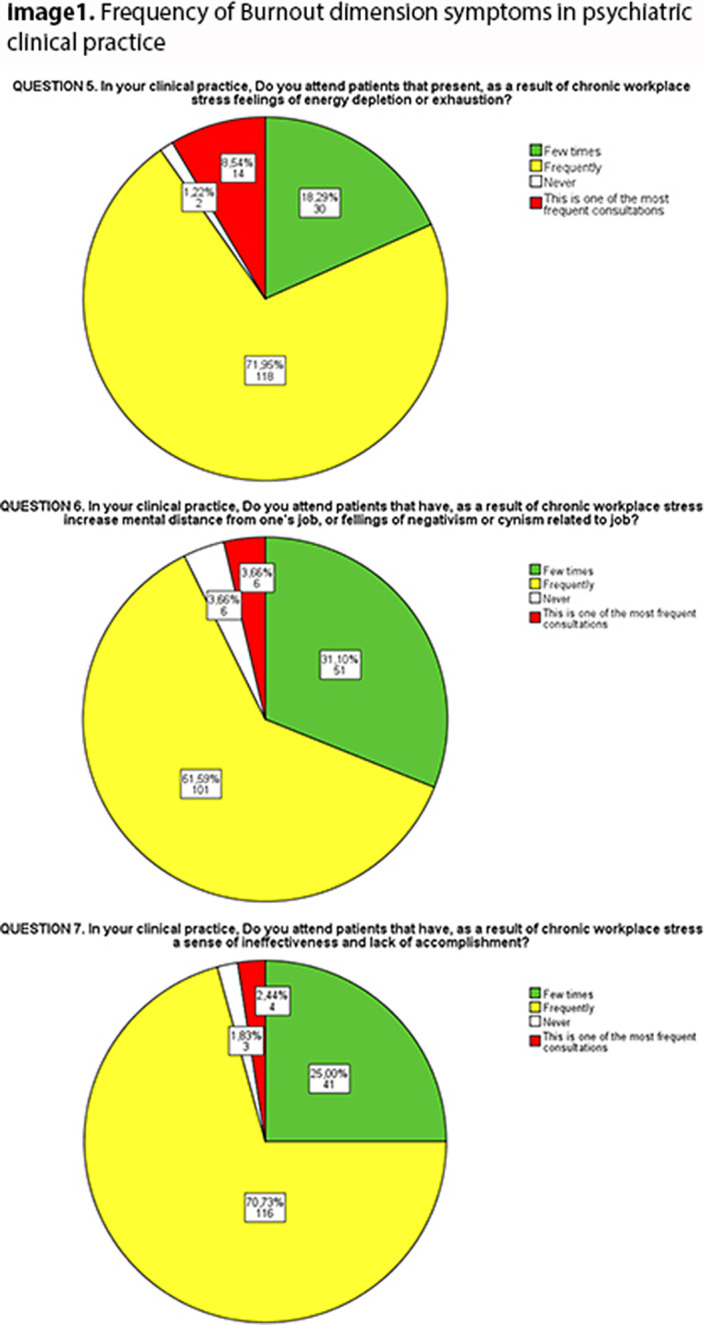

**Image 2:**

**Figure:**
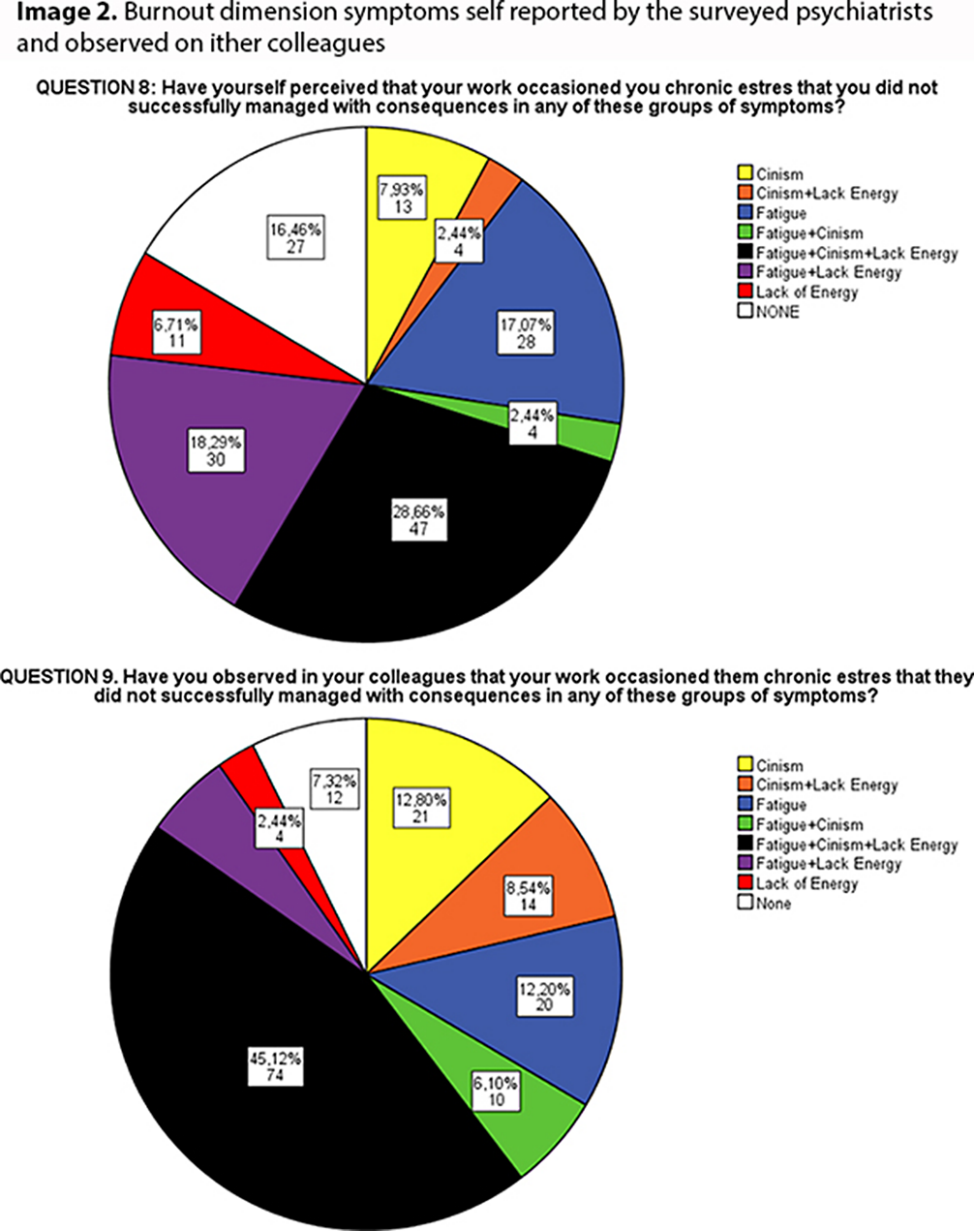

**Image 3:**

**Figure:**
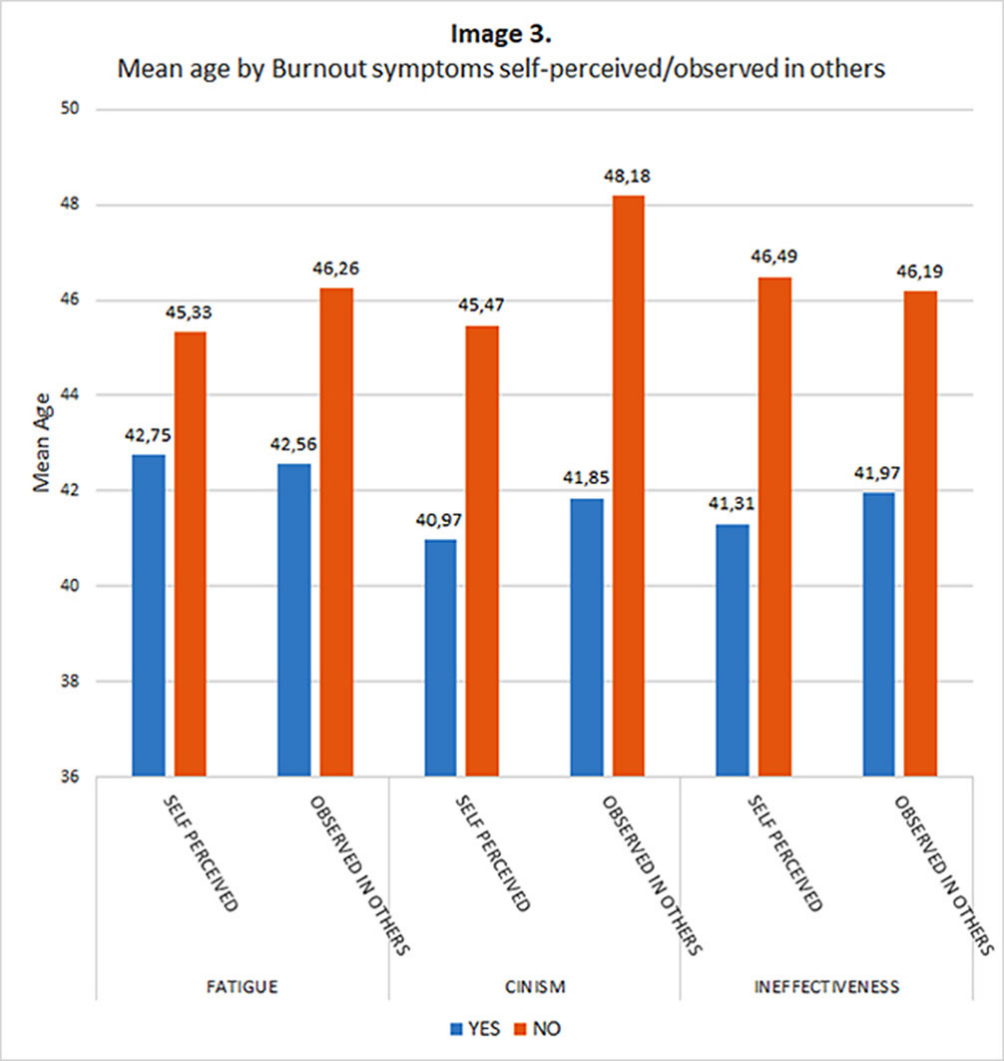

**Conclusions:**

Psychiatrists’ concept of burnout is diverse but the main construct is convergent with ICD definition, not a medical illness but a condition related to work.

The three classic dimensions of burnout are common in clinical conditions and also in the laboral environment of psychiatrists themselves. Psychiatrists tend to recognized more easily burnout in other colleagues, particularly cinism symptoms. Cinism and ineffectiveness appear to be related to younger age that can be associated to an imbalance between work demands and individual resources.

These results highlight the challenge of preventing, detecting and addressing burnout syndrome in psychiatric services.

**Disclosure of Interest:**

None Declared

